# Trimethylamine-N-Oxide (TMAO) as a Rising-Star Metabolite: Implications for Human Health

**DOI:** 10.3390/metabo15040220

**Published:** 2025-03-24

**Authors:** Eugenio Caradonna, Federico Abate, Elisabetta Schiano, Francesca Paparella, Fulvio Ferrara, Emilio Vanoli, Rossana Difruscolo, Vito Maria Goffredo, Bruno Amato, Carlo Setacci, Francesco Setacci, Ettore Novellino

**Affiliations:** 1 Integrated Laboratory Medicine Services, Centro Diagnostico Italiano S.p.A., 20011 Milan, Italyfulvio.ferrara@cdi.it (F.F.); 2 Department of Environmental, Biological and Pharmaceutical Sciences and Technologies, University of Campania Luigi Vanvitelli, Via Vivaldi 43, 81100 Caserta, Italy; federico.abate@unicampania.it; 3 Inventia Biotech-Healthcare Food Research Center S.r.l., Strada Statale Sannitica KM 20.700, 81020 Caserta, Italy; elisabettaschiano@inventiabiotech.com; 4 School of Nursing, Cardiovascular Diseases, University of Pavia, 27100 Pavia, Italy; emivano@gmail.com; 5 Faculty of Medicine, University of Bari, 70121 Bari, Italy; rossanadifruscolo@gmail.com; 6 Department of Interdisciplinary Medicine, Università Degli Studi di Bari, 70124 Bari, Italy; vitogoffredo93@gmail.com; 7 Department of Public Health, Università Degli Studi di Napoli Federico II, 80138 Naples, Italy; bruno.amato@unina.it; 8 Vascular and Endovascuar Surgery Unit, “Le Scotte” Hospital of Siena, Department of Medicine, Surgery and Neuroscience, University of Siena, 53100 Siena, Italy; carlo.setacci@unisi.it; 9 Vascular Surgery Unit, Università degli Studi di Enna “Kore”, 94100 Enna, Italy; francesco.setacci@unikore.it; 10 Department of Medicine and Surgery, Catholic University of the Sacred Heart, 00168 Rome, Italy; ettore.novellino@unicatt.it

**Keywords:** trimethylamine-N-oxide (TMAO), gut microbiota, cardiovascular diseases, atherosclerosis, endothelial dysfunction, neurodegenerative diseases

## Abstract

The intestinal microbiota, hosting trillions of microorganisms that inhabit the gastrointestinal tract, functions as a symbiotic organism that plays a crucial role in regulating health by producing biologically active molecules that can enter systemic circulation. Among them, trimethylamine-N-oxide (TMAO), an organic compound derived from dietary sources and microbial metabolism, has emerged as a critical biomarker linking diet, the gut microbiota, and the host metabolism to various pathological conditions. This comprehensive review highlights TMAO’s biosynthesis, physiological functions, and clinical significance, focusing on its mechanistic contributions to cardiovascular and neurodegenerative diseases. Notably, TMAO-mediated pathways include endothelial dysfunction, inflammation via NLRP3 inflammasome activation, and cholesterol metabolism disruption, which collectively accelerate atherosclerosis and disease progression. Nonetheless, this work underscores the innovative potential of targeting TMAO through dietary, nutraceutical, and microbiota-modulating strategies to mitigate its pathological effects, marking a transformative approach in the prevention and management of TMAO-related disorders.

## 1. Introduction

Trimethylamine (TMA) (C_3_H_9_N) is a small, nitrogen-containing organic compound classified as a tertiary amine. It is a volatile, water-soluble molecule with a strong fishy odor that is commonly found in marine organisms. TMA is produced by the gut microbiota from dietary precursors such as choline, carnitine, lecithin, gamma-butyrobetaine, and phosphatidylcholine [1]. These compounds are found in foods such as red meat; saltwater fish; eggs; dairy products; and some fruits, vegetables, and cereals. In the gut, specific bacterial enzymes convert these precursors into TMA, which is then absorbed into the bloodstream. Once in circulation, TMA is transported to the liver, where it undergoes oxidation to trimethylamine-N-oxide (TMAO), primarily by flavin-containing monooxygenase 3 (FMO3), with a minor contribution from FMO1. While TMAO formation predominantly occurs in the liver, recent studies suggest that certain bacterial species in the gut may also contribute to its production [2,3].

The intestinal microbiome is essential for metabolizing organic substances, substantially influencing the health and disease states of the host. This intricate microorganism ecosystem processes compounds derived from both diet and host, generating metabolites that affect various physiological functions. These byproducts can influence energy metabolism, immune system responses, and even the effectiveness of medications, highlighting the microbiome’s fundamental importance to human well-being [4]. For instance, phytoestrogens are plant-derived compounds that mimic endogenous estrogens, interacting with estrogen receptors to provide potential health benefits, including protection against cardiovascular diseases, cancer, osteoporosis, and menopausal symptoms. These compounds are metabolized by intestinal bacteria into bioactive metabolites, such as equol, urolithins, and enterolignans, which can be enhanced by probiotics to increase their beneficial effects. The gut microbiota’s role in metabolizing phytoestrogens suggests that these compounds can modulate the microbial community structure, potentially affecting the production of TMAO from dietary choline and carnitine [5].

In recent years, TMAO has emerged as a key metabolite at the intersection of diet, the gut microbiota, and human metabolism. While it serves essential physiological functions, numerous pieces of evidence have linked elevated circulating TMAO levels to the development of cardiovascular disease, kidney dysfunction, metabolic disorders, and neurodegenerative conditions. Its ability to influence endothelial function, lipid metabolism, inflammation, and platelet reactivity suggests that it may act as a key mediator in the pathogenesis of these conditions, making it not only a potential biomarker of disease risk, but also a novel target for therapeutic interventions. This review aims to provide a comprehensive analysis of the current knowledge regarding TMAO, exploring its biosynthesis, physiological functions, and pathological implications. By examining its involvement in cardiovascular and neurological disorders alongside potential strategies for modulating its levels, this work seeks to shed light on the complex duality of TMAO as both a physiological osmolyte and a contributor to disease. Additionally, herein, we discuss emerging strategies to regulate its levels through dietary interventions, gut microbiota modulation, and nutraceutical approaches.

### 1.1. Biosynthesis of TMAO

TMAO is a water-soluble molecule that plays diverse physiological roles. It acts as an osmolyte in marine organisms, stabilizing proteins under high-pressure environments, and has been implicated in various human metabolic pathways [6]. The synthesis of TMAO occurs via a two-step metabolic pathway. First, the gut microbiota converts dietary nutrients into TMA. Specific bacterial strains, including members of the genera *Escherichia*, *Clostridium*, and *Anaerococcus*, are known to possess the enzymatic machinery required for TMA production [7]. Precursors of TMA include choline (abundant in eggs, meat, and dairy products), L-carnitine (primarily found in red meat), and betaine (present in plant-based foods such as wheat and spinach) [3].

TMA and the above precursors can be converted into TMAO through specific bacterial enzymes. The known enzymatic pathways are choline TMA lyase (cutC/D), carnitine monooxygenase (cntA/B), betaine reductase, and TMAO reductase [1]. The CutC/D system is a glycyl radical enzyme complex widely present in the gut microbiome, particularly in members of *Firmicutes*, *Actinobacteria*, and *Proteobacteria* [8]. In contrast, the CntA/B pathway is more characteristic of *Gammaproteobacteria*, predominantly found in species such as *Escherichia coli* and *Acinetobacter baumannii* [1]. Research using metagenomic screening has demonstrated that cutC genes are almost universally present in human gut microbiomes, while cntA genes appear in a smaller proportion of individuals [9]. Although TMA-producing bacteria constitute less than 1% of the overall gut microbiota, their metabolic processes are still capable of markedly affecting circulating TMAO levels [10].

Other genes are also closely related to cntA and cntB, such as yeaW and yeaX, which encode a monooxygenase and a reductase, respectively. The functional domain of the YeaW monooxygenase exhibits nearly identical characteristics to that of the cntA monooxygenase. In conjunction with the yeaX reductase, it facilitates the production of TMA through a process that utilizes γ-butyrobetaine (γBB) as an intermediary compound [11]. Bacteria with the *gbu* gene cluster play a role in producing TMA from L-carnitine metabolism. Notable examples include *Emergencia timonensis*, *Agathobaculum desmolans*, and *Intestinibacillus* sp. [12], which are particularly important in converting γBB into TMA, a precursor of TMAO. However, recent research indicates that the diversity of TMA-producing microbiota, both taxonomically and functionally, is likely broader than currently recognized, with many yet-to-be-cultured bacterial species potentially involved in this process.

TMA is also obtained from reducing the TMAO present in the diet by gut flora. *Enterobacteriaceae*, particularly *Escherichia coli* and *Klebsiella pneumoniae*, are predominantly responsible for the conversion of TMAO into TMA in the intestinal environment [13]. This bidirectional metabolism highlights the complex interplay between host and microbial factors in regulating circulating TMAO levels, which have been linked to cardiovascular disease (CVD), metabolic disorders, and chronic kidney disease (CKD) [14]. Glycine betaine serves as the precursor for TMA production. The enzyme responsible for the conversion of glycine betaine to acetate and TMA is encoded by the grdH gene, which produces a glycine betaine reductase. In this process, the acetate molecule undergoes oxidation, thereby generating the electrons necessary for reducing betaine [15].

### 1.2. TMAO Metabolism

**Figure 1:**
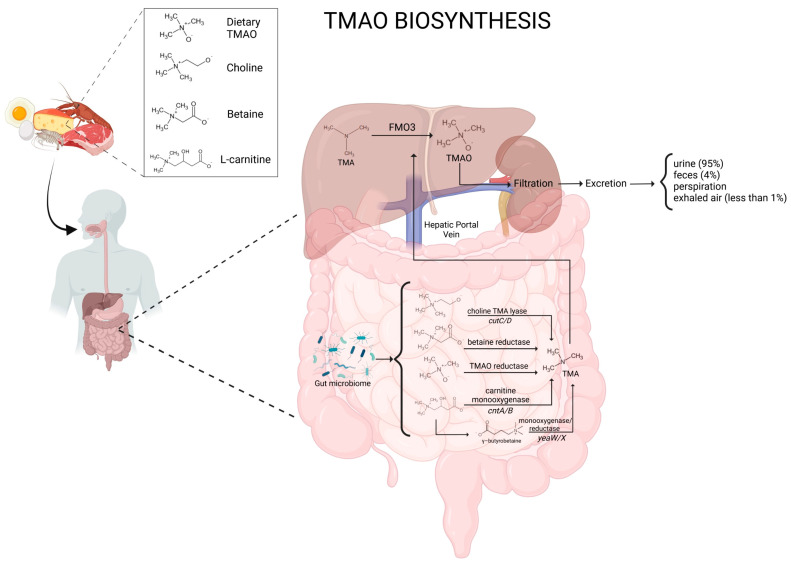
Schematic representation of TMAO biosynthesis and metabolism.

Once TMA enters the bloodstream, it is transported to the liver, where flavin-containing monooxygenases (FMOs), particularly FMO3, catalyze its oxidation into TMAO [16]. The enzymatic activity of FMO3 is 10-fold greater than that of FMO1, which is mainly expressed in the kidneys and intestine [17,18]. Nonetheless, recent research highlights that TMAO is not only formed systemically, but also produced locally within the gut by bacterial FMO-like enzymes [19]. Interestingly, genetic variations and hepatic conditions can affect the efficiency of TMAO metabolism, leading to interindividual differences in its plasma levels [20]. Humans have five functional genes for FMO [21], and variations in FMO3 expression among individuals are likely attributable to genetic and physiological factors. Genetic polymorphisms in the FMO3 gene can influence the activity of the enzyme. Certain variants may result in reduced FMO3 activity, consequently leading to decreased levels of TMAO [22]. The dysbiosis of oral microbiota may potentially alter the expression of FMO3, resulting in an increase in the production of TMAO, thereby linking oral health to systemic health through the translocation of bacteria in the bloodstream [23]. As an example, *Porphyromonas gingivalis*, an oral pathogen, has been shown to upregulate the expression of FMO3 in the liver [23]. TMAO production is lower in males compared to females due to the inhibitory effect of testosterone on FMO3 [17]. Recent evidence suggests that TMAO is also produced in human and mouse aorta endothelial cells [24]. TMAO, which is transported to the tissues for accumulation as an osmolyte, or more frequently cleared by the kidneys, is subsequently excreted, primarily in urine at a 3:95 TMA–TMAO ratio within 24 h. Alternative routes of TMAO excretion include perspiration, feces (4%), exhaled air (less than 1%), and other bodily secretions [6]. A schematic representation of TMAO biosynthesis and metabolism is reported in Figure 1.

### 1.3. Physiological Functions of TMAO

Structurally, TMAO comprises a trimethylated amine group bonded to an additional oxygen atom, making it a highly water-soluble compound. This molecule is strongly polar, basic, an excellent hydrogen bond acceptor (even better than water itself), and a good oxidizing agent [25]. Beyond human health, TMAO is an important metabolic byproduct in mammals and serves essential physiological functions in marine species [26]. In mammals, a TMAO value below 4 micromoles/L is generally considered a beneficial indicator. A key physiological role of TMAO is its function as an osmolyte, which helps regulate cell volume and stabilize proteins from denaturation under stressful conditions, such as high salinity, high pressures, or temperature fluctuations [27]. Notably, TMAO is recognized for its potent chaperone activity as it enhances protein stability by interacting with their hydration shells, thereby preventing aggregation and misfolding [28]. This function is particularly advantageous in addressing misfolded proteins associated with protein conformational diseases, as TMAO can help restore the activity of specific mutant proteins, potentially aiding in their proper folding. Additionally, its osmotic properties play a crucial role in marine organisms, like fish and crustaceans, enabling them to adapt to fluctuating salinity levels in their environment [29].

In mammals, TMAO is predominantly studied from the perspective of metabolic cycles and cardiovascular health, playing a significant role in lipid metabolism, inflammatory responses, and endothelial function [14]. Research suggests that TMAO contributes to cholesterol transport regulation and bile acid metabolism, indicating a role in maintaining lipid balance. Nevertheless, elevated TMAO levels have been correlated with an increased risk of atherosclerosis and CVDs [30]. In this context, studies indicate that TMAO may enhance vascular inflammation, promote platelet hyperreactivity, and accelerate foam cell formation, all of which are key factors in the development of atherosclerosis [31]. Specifically, a meta-analysis conducted in 2017 revealed that higher circulating TMAO levels were associated with a 23% increased risk of cardiovascular events and a 55% higher risk of mortality [32]. Nonetheless, TMAO plays a crucial role in the progression of neurodegenerative diseases, such as Parkinson’s disease (PD) and Alzheimer’s disease (AD) [33,34]. Its intricate interactions with cellular metabolic pathways may also significantly contribute to tumor development [35]. For instance, elevated TMAO levels have been linked to reduced survival rates in patients with colorectal cancer [36]. Based on this evidence, the quantification of TMAO in the bloodstream holds particular relevance for both the prevention and treatment of these conditions, as its involvement in the pathophysiology of various metabolic disorders is now well established.

## 2. TMAO’s Cellular Effect

### 2.1. Arteriosclerosis

Conventional and unconventional risk factors contribute to atherosclerosis, a chronic inflammatory condition affecting blood vessels. TMAO facilitates the progression of atherosclerosis through multiple complex cellular mechanisms. TMAO enters endothelial cells through endothelial TMAO transporter 1 (ETT1) by inducing the activation of the cytosolic multiproteic complex inflammasome NOD-like receptor protein 3 (NLPR3) and promoting endothelial to mesenchymal cell transition [37]. The activation of NLRP3 is one of the key factors of atherosclerosis and is related to many chronic diseases [38]. TMAO activates NLRP3 through multiple mechanisms and facilitates the onset of atherosclerosis. The activation of NLRP3 via inflammation is partly mediated by the TMAO-induced inhibition of the mitochondrial reactive oxygen species (mtROS) signaling pathway sirtuin3-superoxide dismutase-2 (SIRT3-SOD2). SIRT3 can directly bind to and deacetylate manganese superoxide dismutase 2 (SOD2), resulting in increased SOD2 activity and exerting significant effects on mtROS homeostasis and NLRP3 inflammation [39]. The inhibition of the SIRT3-SOD2 pathway increases the concentration of ROS and induces the dissociation of thioredoxin (TRX)-interacting protein (TXNIP) from thioredoxin in a ROS-sensitive manner, subsequently enabling TXNIP to bind to NLRP3 [40]. Xiaolei Sun et al. demonstrated that in human umbilical vein endothelial cells, trimethylamine-N-oxide (TMAO) induces inflammation and endothelial dysfunction by activating the inflammasome thioredoxin-interacting protein-associated NLRP3 (TXNIP-NLRP3) [41].

The sirtuin family (1–7) nicotinamide adenine dinucleotide-dependent class III histone deacetylases regulates important cellular functions such as metabolism, apoptosis, cell cycles, and gene transcription. SIRT3, located in the mitochondria, is an important member of the Sir2 family, the essential action of which is regulating mtROS levels [42]. SIRT3 plays a crucial role in cardiovascular disease and represents a potential therapeutic target in heart failure and peripheral arterial disease [43,44]. The activation of sirtuin e 1 and 6 reduces inflammation and oxidative stress [45]. Elevated concentrations of TMAO can expedite endothelial cell senescence through the inhibition of SIRT1 activity [46].

The mitogen-activated protein kinase (MAPK) protein complex contributes to the pathogenesis of atherosclerosis through its regulation of endothelial and smooth muscle cell proliferation and migration within the vessel wall [47]. TMAO activates the MAPK/JNK (c-Jun N-terminal kinase) pathway through CD36 upregulation, leading to enhanced foam cell formation and atherosclerotic plaque progression [48]. CD36 is a widely expressed glycoprotein that functions as a scavenger receptor and facilitates the uptake of oxidized LDL (ox-LDL), a process that is essential for foam cell formation [49]. TMAO enhances the expression of scavenger receptors (CD36, CD 4, and SR-A1) in macrophages, resulting in an increase in cholesterol within macrophages and the formation of foam cells in atherosclerotic lesions [50].

TMAO has been demonstrated to disrupt lipid metabolism, with significant correlations between TMAO levels and blood lipids, such as triglycerides and high-density lipoprotein cholesterol (HDL-C) [51]. Experimental studies indicate that TMAO can impede cholesterol metabolism, contributing to the pathogenesis of thrombosis and atherosclerosis [52]. Mechanistic studies suggest that TMAO affects cholesterol metabolism by altering bile acid synthesis and feedback regulation, as well as influencing the expression of genes involved in cholesterol transport and metabolism [53]. TMAO induces alterations in cholesterol metabolism, which may contribute to atherosclerotic plaque formation. It enhances macrophage cholesterol uptake through the upregulation of receptors such as CD36 and scavenger receptor A [52]. Furthermore, TMAO disrupts bile acid synthesis and transintestinal cholesterol export, potentially leading to increased cholesterol levels in the liver and plasma. Bile acid synthesis is the primary mechanism by which the body eliminates excess cholesterol. TMAO disrupts this process by suppressing the expression of key enzymes involved in bile acid production, such as CYP7A1 and CYP27A1 [14,54]. This results in decreased cholesterol elimination and increased cholesterol levels in the liver, which can subsequently re-enter the bloodstream and contribute to atherosclerosis. These mechanisms suggest that TMAO plays a significant role in promoting atherosclerosis through its effects on cholesterol metabolism and foam cell formation in atherosclerotic plaques.

Reverse cholesterol transport (RCT) is a critical process for the removal of cholesterol from arteries and its subsequent transportation to the liver for excretion [55]. TMAO impedes this pathway, resulting in cholesterol accumulation in the arteries [3]. Koeth et al. demonstrated how TMAO significantly reduces the reverse transport of cholesterol [54].

### 2.2. Inflammation

Chronic inflammation has been implicated in a wide range of diseases, with more than 50% of all deaths worldwide attributed to chronic inflammatory diseases, such as cardiovascular disease, type 2 diabetes, neurodegenerative diseases, cancer, metabolic diseases, gastrointestinal diseases, and respiratory diseases. Elevated levels of TMAO enhance the expression of inflammatory cytokines, such as tumor necrotic factor-α (TNF-α) and interleukin-6 (IL-6), which facilitate the migration and accumulation of macrophages in the arterial wall [13]. As previously stated, TMAO can activate the NLRP3 inflammasome, which plays a key role in innate immunity. NLRP3 inflammasome activation is mediated by the interaction of TMAO with pyrin domains (PYDs) of the adaptor protein ASC, which in turn recruits pro-caspase-1 and induces its self-activation. Activated caspase-1 promotes the maturation and release of pro-inflammatory cytokines, such as interleukin-1β (IL-1β) and interleukin-18 (IL-18) [56,57]. The apoptosis-associated speck-like protein (ASC), which contains a caspase recruitment domain (CARD), serves as a key adaptor protein in inflammasome activation. In turn, NLRP3 can trigger ROS in multiple cell types, such as macrophages and endothelial cells [41]. TMAO reduces the expression of anti-inflammatory cytokines, such as IL-10 [58].

TMAO has the ability to induce oxidative stress in cells by promoting the production of reactive oxygen species (ROS). An overabundance of ROS can be detrimental, leading to damage to essential cellular components, such as DNA, proteins, and lipids. Additionally, this overabundance of ROS can stimulate pro-inflammatory signaling pathways, such as the nuclear factor kappa-light-chain-enhancer-activated B cell (NF-κB) pathway [7]. TMAO has been shown to increase the activity of NADPH oxidase [30], an enzyme that generates ROS as part of its normal function. This upregulation can contribute to an overall increase in ROS production and oxidative stress within cells.

TMAO can contribute to endoplasmic reticulum (ER) stress, a cellular reaction to disruptions in protein folding and processing. This stress response can, in turn, elevate ROS production due to imbalances in cellular homeostasis [24,59]. Additionally, TMAO has been shown to activate the protein kinase R-like endoplasmic reticulum kinase (PERK) signaling pathway, one of the three major pathways involved in the ER stress response [60]. PERK is a protein kinase that phosphorylates eukaryotic translation initiation factor 2α (eIF2α), resulting in the inhibition of global protein synthesis and the activation of transcription factors that regulate the expression of genes involved in the endoplasmic reticulum stress response. TMAO activates the PERK pathway and induces alterations in synaptic and neuronal plasticity. This mechanism may contribute to age-related cognitive decline and the development of neurodegenerative disorders such as Alzheimer’s disease [61].

The existence of a dynamic interplay among inflammation, the nervous system, and the immune system is a well-established phenomenon with rapidly expanding research. This interaction is initiated through the release of factors such as cytokines, histamine, and lipids (e.g., prostaglandins and leukotrienes) by immune cells during injury and/or infection, subsequently affecting sympathetic activity. This mechanism creates a positive feedback loop in which the sympathetic nervous system (SNS) promotes inflammation, which subsequently enhances the activation of sympathetic neural endings [62]. Notably, experimental studies on the ischemic heart provide significant support for the stimulatory effect of inflammation on the SNS [63]. In one investigation, interleukin-1β (IL-1β) injected into the left stellate ganglion (LSG) resulted in a decrease in the ventricular effective refractory period, action potential duration (APD) 90, and ventricular arrhythmia (VA) occurrence, as well as an increase in sympathetic indices of heart rate variability (HRV) and LSG activity in both normal and ischemic hearts. This observation was corroborated by the elevated mRNA expression of pro-inflammatory cytokines; the increased protein expression of c-fos, nerve growth factor, and neuropeptide Y in the LSG; and the decreased neuronal nitric oxide synthase expression. Consistent with these findings, LSG TMAO administration significantly enhanced LSG function and activity, reduced the effective refractory period, and exacerbated ischemia-induced VA. pro-inflammatory markers were also significantly upregulated by TMAO. Furthermore, evidence suggests that TMAO contributes to sympathetic hyperactivity in aging by downregulating P2Y12R in microglia and increasing inflammation in the PVN [64].

This experimental background was clinically confirmed in ischemic heart failure patients in which SIRT1, apoA1, TMAO, and NGF serum levels were found to be abnormally expressed and closely related to cardiac function [65].

Gut-innervating DRG neurons play a crucial role in regulating the intestinal barrier and microbiota, which are key factors in TMAO production. Maintaining a healthy gut barrier and microbiota can help to reduce TMAO levels and potentially mitigate its associated health risks [66]. Elevated levels of TMAO induce a state of chronic inflammation through a complex interplay of mechanisms.

### 2.3. Endothelial Progenitor Cells

The abnormal expression of endothelial progenitor cells (EPCs) and endothelial dysfunction are among the initial indicators of atherosclerosis [67]. Moreover, a reduction in the number of EPCs is associated with an increased risk of mortality in patients with stable coronary disease and vasculopathy [68]. EPCs play a key role in repairing ischemic damage [69]. Furthermore, a reduced level of EPCs is associated with an increased risk of cardiovascular events and mortality [70]. TMAO levels are associated with increased oxidative stress and decreased numbers of circulating endothelial progenitor cells [71].

TMAO appears to impair neovascularization through the inactivation of the Akt/eNOS and MAPK/ERK signaling pathways in EPCs [72]. TMAO has been demonstrated to enhance the expression of microRNA-221 (miR-221) in EPCs. MiR-221 is recognized for its inhibition of angiogenesis and its contribution to endothelial dysfunction. TMAO can indirectly affect EPCs by influencing the release of exosomes from hepatocytes. These TMAO-treated exosomes can be internalized by EPCs and impair their function, thereby further contributing to endothelial dysfunction [73]. These signaling pathways play a vital role in regulating cell survival, growth, and differentiation. Any disruption in these mechanisms can severely impair the EPC function. Although the association between TMAO and EPC dysfunction is becoming increasingly evident, further investigations are necessary to elucidate the underlying mechanisms and explore potential therapeutic interventions to mitigate these effects.

The data obtained in our laboratory demonstrated that TMAO exerts a significant inhibitory effect on endothelial nitric oxide synthase (eNOS), impairing nitric oxide (NO) production and vascular function through multiple mechanisms. TMAO directly competes with L-arginine at the catalytic site of eNOS, effectively reducing NO synthesis and acetylcholine (Ach)-induced relaxation in murine and rat aortas. Furthermore, TMAO docking at the L-arginine binding site disrupts its interaction with L-arginine, with the N-oxide group chelating Fe^2+^ and the positively charged nitrogen forming cation–π interactions, further preventing NO production. Additionally, elevated TMAO levels promote eNOS uncoupling, leading to ROS generation instead of NO synthesis, thereby exacerbating oxidative stress and endothelial dysfunction. TMAO-mediated inhibition of eNOS leads to the release of pro-inflammatory cytokines while also hindering neovascularization and tissue regeneration. This occurs through the suppression of endothelial progenitor cell migration and differentiation via the AKT/eNOS pathway. Consequently, TMAO-induced eNOS inhibition compromises vasorelaxation, contributing to vascular dysfunction (in the published results).

### 2.4. Monocyte–Macrophage Axis

The monocyte–macrophage axis constitutes a complex and dynamic system characterized by intricate interactions between diverse subsets and phenotypes. Monocytes and macrophages engage in communication with other immune cells, including lymphocytes and dendritic cells, to orchestrate immune responses. Furthermore, they interact with non-immune cells, such as endothelial cells and fibroblasts, to regulate tissue homeostasis and repair [74]. The dysregulation of the monocyte–macrophage axis is implicated in various diseases, including infections, autoimmune diseases, and cardiovascular diseases [75].

While studies directly examining the impact of TMAO on monocyte subsets remain limited, existing research indicate a complex interplay between TMAO, monocytes, and macrophages, with potential implications for inflammation and cardiovascular disease. TMAO has been associated with increased levels of pro-inflammatory intermediate monocytes (CD14++CD16+) [76]. TMAO can induce endoplasmic reticulum (ER) stress in macrophages, leading to the activation of Toll-like receptor 4 (TLR4). TLR4 activation triggers the production of pro-inflammatory cytokines, contributing to inflammation and potentially exacerbating atherosclerosis [77].

Intermediate monocytes have been identified as an independent predictive factor for coronary artery disease, peripheral arterial disease, and chronic kidney disease [78]. As previously described, TMAO promotes the transformation of macrophages into foam cells, which are significant contributors of atherosclerosis. This mechanism leads to an increased uptake of oxidized LDL cholesterol and a reduction in cholesterol efflux, ultimately causing lipid buildup in macrophages and contributing to the development of atherosclerotic plaques [79]. TMAO can indirectly influence monocytes and macrophages by promoting endothelial dysfunction. This dysfunction involves an increased expression of adhesion molecules such as VCAM-1, which facilitates monocyte adhesion to the endothelium and their subsequent migration into tissues, where they differentiate into macrophages and contribute to inflammation [30].

### 2.5. Platelets

TMAO enters platelets and facilitates the release of calcium ions (Ca^2+^) from intracellular stores. Calcium serves as a critical secondary messenger in platelet activation, and its elevated levels promote platelet aggregation and thrombus formation. Zhu et al. demonstrated that TMAO induces submaximal stimulus-dependent platelet hyperactivity from multiple agonists through increased intracellular Ca^2+^ release [80]. Elevated calcium levels can activate phospholipase C (PLC), an enzyme that plays a crucial role in generating IP3 [37]. IP3 is another signaling molecule involved in platelet activation [81]. This further amplifies the platelet’s response to stimuli [80].

TMAO has been demonstrated to contribute to platelet hyperreactivity and thrombosis through the augmentation of the expression and activity of tissue factor, a protein implicated in the coagulation cascade [82]. TMAO’s interaction with platelets disrupts metabolic activation and reduces their responsiveness to clopidogrel, a process mediated by NOX-dependent ROS/Nrf2/CES activation [83]. The effects of TMAO on various cell types and the related molecular mechanisms are reported in Table 1.

## 3. Clinical Implications of TMAO

### 3.1. Atherosclerosis

The development of atherosclerosis starts with the activation of endothelial cells and the accumulation of cholesterol within the vessel walls. This process leads to the transformation of macrophages into foam cells through the phagocytic uptake of lipid particles [88]. The oxidation of these lipids triggers several events: cholesterol crystallization; inflammasome activation; and the production of pro-inflammatory cytokines, including TNF-α and interleukin-1B (IL-1B). This cascade of events culminates in vessel narrowing, collectively contributing to cardiovascular complications, which remain the leading cause of mortality worldwide [89].

Various studies have established that TMAO is also involved in the progression of atherosclerosis through different mechanisms, including the promotion of vascular inflammation and reduced reverse cholesterol transport, as mentioned in Section 2.1 of this review [90]. From a clinical point of view, several human studies have investigated the relationship between circulating TMAO levels and atherosclerosis, providing mixed but significant findings [79]. Specifically, a prospective cohort study involving 671 patients showed that elevated TMAO levels are associated with a greater risk of cardiovascular events in individuals with a history of ischemic stroke. However, that study had some limitations, such as a one-year follow-up period and data collection from a single research center [76]. Similarly, a two-year controlled cohort study involving 100 patients found a strong association between elevated plasma TMAO levels and the onset of new atherosclerosis, as well as plaque rupture in cases of very late stent thrombosis (VLST) [91]. Another case–control study with 2595 participants performed a 24 h carnitine challenge test, showing that individuals with high plasma TMAO levels exhibit a strong association between excessive L-carnitine intake and increased cardiovascular disease (CVD) and adverse event risk [54]. Li et al. conducted a study on 179 patients with ST-elevation myocardial infarction (STEMI) and found a positive correlation between TMAO levels and the presence of calcification in intimal lesions [92]. Similarly, the link between serum TMAO levels and early-stage atherosclerosis was explored in a cohort of 220 participants, where carotid intima–media thickness (cIMT) served as a marker for subclinical atherosclerosis. The results indicated a significant positive correlation between elevated TMAO levels and increased cIMT (r = 0.26, *p* < 0.0001). Notably, even after adjusting for variables such as age, sex, and visceral fat, TMAO remained an independent predictor of cIMT [93].

Furthermore, a study carried out by Bao et al. examined the relationship between plasma TMAO levels and coronary atherosclerotic burden in a cohort of 429 newly diagnosed coronary heart disease (CHD) patients. The results showed a positive correlation between TMAO levels and SYNTAX scores, a tool used to score the complexity of coronary artery disease in clinical practice, with higher TMAO concentrations associated with greater atherosclerotic burden (*p* = 0.006) [94]. Noteworthily, a systematic review of 1622 participants with an average follow-up of 4.95 years supported the link between high TMAO concentrations and increased mortality risk [95]. To strengthen this evidence, a meta-analysis including 31,230 individuals and a systematic review analyzing 15,662 and 13,944 participants, with an average follow-up of 4.3 ± 1.5 years, found a dose-dependent positive correlation between plasma TMAO levels, cardiovascular events, and mortality [96]. On the other hand, a case–control study with a 24-week follow-up revealed that oral L-carnitine supplementation increased plasma TMAO levels, but had no significant impact on lipid profiles or other cardiovascular markers. However, this study only monitored cardiac markers for a short 24-week period, limiting long-term conclusions [97].

TMAO also represents one of the most important risk factors for major adverse cardiovascular events (MACEs). In this context, Wilson Tang et al. conducted an 8-year follow-up study on a middle-aged population as part of the EPIC-Norfolk Study, reporting a significant association between TMAO blood levels and the development of coronary artery disease (CAD) [98]. Similarly, Heianza et al. observed the same correlation in a cohort of healthy women monitored over a 10-year period [99]. Xinmin S. Li et al. demonstrated that, in patients with acute coronary syndrome, TMAO is associated with a significant increase in the incidence of MACEs during follow-up (20 days to 6 months) [100]. Elevated TMAO levels are also associated with higher mortality in patients with stable CAD and optimal medical therapy during a 5-year follow-up [101]. Based on this evidence, clearly, assessing TMAO blood levels could be valuable in identifying patients with a high atherosclerotic burden, and may serve as a predictive biomarker for cardiovascular risk, even in asymptomatic individuals.

### 3.2. Heart Failure

Extensive research has demonstrated a strong correlation between TMAO and an increased risk of heart failure (HF), especially in individuals with pre-existing cardiovascular conditions. TMAO may contribute to HF by inducing an inflammatory response characterized by increased levels of cytokines (IL-6, IL-1, and TNF-α), as well as oxidative stress and endothelial dysfunction. As previously described, these effects are largely mediated by the activation of the NLRP3 inflammasome and the inhibition of the SIRT3-SOD2 pathway, leading to increased mitochondrial ROS production and endothelial dysfunction [39].

TMAO has been shown to increase oxygen consumption and alter cardiac metabolism among HF patients [102,103]. In acute HF, the combination of TMAO and N-terminal pro-b-type natriuretic peptide (NT-pro BNP) has been shown to improve risk stratification for one-year mortality [104]. Additionally, TMAO has been identified as a crucial prognostic marker in the progression of HF [105,106]. Another study found that in addition to TMAO, both choline and betaine appeared to contribute to worsening left ventricular diastolic dysfunction. However, after adjusting for cardiopulmonary parameters, only elevated TMAO levels retained a significant adverse prognostic impact [107].

A subsequent analysis in a Norwegian cohort reinforced these findings, demonstrating a correlation between circulating TMAO levels in chronic HF (CHF) patients and the New York Heart Association (NYHA) classification, ischemic etiology, mortality, and post-heart transplant survival rates [108]. In this context, the BIOSTAT-CHF study revealed that elevated TMAO levels are associated with increased mortality rates, independent of ongoing treatment [109]. For instance, Li et al. demonstrated that loop diuretics (LDs) reduce the renal elimination of TMAO, suggesting that doses of furosemide exceeding 40 mg may be correlated with higher hospitalization and mortality rates [110], serving as an independent predictor of three-year mortality [111].

### 3.3. Peripheral Vascular Disease

Emerging evidence indicates that TMAO plays a significant role in the progression of peripheral vascular disease (PVD), a condition characterized by reduced blood flow to the extremities due to atherosclerotic occlusions. As previously outlined, elevated TMAO levels have been associated with endothelial dysfunction, vascular inflammation, and impaired microcirculation, all of which contribute to ischemic symptoms in PVD patients [112]. Mainly, TMAO promotes oxidative stress and accelerates endothelial senescence by downregulating SIRT1, a key regulator of vascular homeostasis [46]. Additionally, TMAO activates the NLRP3 inflammasome, triggering the release of pro-inflammatory cytokines, exacerbating endothelial dysfunction, and impairing NO-mediated vasodilation [39]. Another crucial mechanism involves the negative impact of TMAO on EPCs, which play a pivotal role in vascular repair and angiogenesis [67]. Furthermore, TMAO has been linked to increased platelet hyperreactivity and higher thrombotic risk, which are major contributors to critical limb ischemia (CLI), the most severe form of PVD [80,82].

From a clinical point of view, numerous studies have demonstrated a direct correlation between high plasma TMAO levels and increased PVD severity, adverse post-revascularization outcomes, and higher amputation rates [113,114]. Specifically, a study conducted by Roncal et al. demonstrated that patients with CLI exhibit significantly higher TMAO levels compared with those with intermittent claudication, and this elevation is associated with an increased risk of mortality, independent of traditional cardiovascular risk factors. More specifically, cardiovascular mortality has been linked to plasma TMAO levels exceeding 2.26 µmol, with a reported sensitivity of 62% and a specificity of 76% [115]. Additionally, TMAO negatively impacts angiogenesis and perfusion in patients with PAD, further exacerbating ischemic complications [116]. Studies have also shown that TMAO levels tend to be higher in diabetic patients and individuals undergoing prolonged statin therapy, potentially accelerating atherosclerosis progression [117]. Given these findings, TMAO has the potential to serve as a novel biomarker for disease progression and prognosis in PVD patients.

### 3.4. Hypertension

As discussed earlier, high TMAO levels have been linked to endothelial dysfunction, characterized by reduced NO availability and increased arterial stiffness, both of which contribute to hypertension [118]. Additionally, a study conducted by Jiang et al. found that TMAO exacerbates hypertension by interacting with the angiotensin II receptor, resulting in increased vascular resistance and blood pressure elevation [119]. Clinical findings have strengthened the link between elevated plasma TMAO and hypertension development. Indeed, longitudinal studies have shown that higher baseline TMAO concentrations are predictive of future hypertension, even after adjusting for conventional risk factors (e.g., age, dietary sodium intake, and obesity) [120]. Additionally, excessively high TMAO concentrations have been correlated with an increased risk of stroke and mortality in hypertensive patients, underscoring its broader impact on vascular health and cerebrovascular events [121]. Moreover, recent findings indicate that angiotensin-converting enzyme (ACE) inhibitors, such as enalapril, may help lower TMAO levels, suggesting a potential mechanism through which these medications exert their antihypertensive effect [122].

## 4. Involvement of TMAO in Neurodegenerative Diseases

### 4.1. Alzheimer’s Disease

TMAO has been linked to cognitive decline and neurodegenerative disorders, including Alzheimer’s disease (AD). Through an integrated analysis of genetic, epigenetic, pathological, and biochemical data, Xu et al. identified a correlation between gut microbiota-derived metabolites, especially TMAO, and AD [123]. The detection of TMAO in cerebrospinal fluid (CSF) confirms its ability to cross the blood–brain barrier (BBB), with approximately 26% passively diffusing within 12 h of perfusion [124]. At physiological levels (0.11 to 6.45 µmol/L) [125], TMAO contributes to BBB stabilization and protection by promoting protein folding and hydration [126]. This effect is attributed to a nuanced mechanism where TMAO acts as a molecular crowder, becoming depleted from the protein surface as the protein assumes its native conformation [33]. Additionally, TMAO interacts more strongly with water molecules than with neighboring TMAO molecules, forming complexes with two–three water molecules. This results in an expanded hydration shell around TMAO, influencing protein hydration and stabilization [127].

However, excessive TMAO concentrations disrupt BBB integrity by inhibiting tight junction proteins, including claudin-5 and zonula occludens-1 [123,126], leading to microangiopathy, impaired cerebral perfusion, and cognitive decline, particularly in aging individuals [124]. Beyond its impact on BBB integrity, TMAO also triggers neuroinflammation by activating the NLRP3 inflammasome, a key protein in AD progression [128]. In the context of neuroinflammation, a key factor is clusterin, a secreted heterodimeric glycoprotein with a molecular weight of 75–80 kDa that represents the second most abundant apolipoprotein associated with AD progression [129]. A critical aspect of TMAO’s involvement in AD is its ability to increase clusterin expression, thereby amplifying neuroinflammatory processes [33]. As highlighted in Section 2.2, TMAO enhances the ER stress response by activating the PERK-eIF2α pathway, which is known to impair synaptic plasticity and neuronal function, processes strongly implicated in AD progression [24]. This ER stress response also contributes to the production of pro-inflammatory mediators such as IL-1β and TNF-α, exacerbating neurodegeneration [59].

Furthermore, TMAO facilitates β-amyloid fibril conversion, tau aggregation [130], and mitochondrial dysfunction [131], contributing to oxidative stress and neuronal degeneration [4].

In support of this evidence, studies on middle-aged and elderly individuals (65 ± 7 years) have reported an inverse correlation between plasma TMAO levels and cognitive performance, particularly in memory and fluid cognition assessments using the NIH Toolbox cognitive battery [132]. Notably, a large-scale study by Vogt et al. examined the association between TMAO and AD by analyzing CSF TMAO concentrations in a diverse cohort. This included individuals diagnosed with clinical AD (*n* = 40), those with mild cognitive impairment (MCI, *n* = 35), and cognitively unaffected participants (*n* = 335). The study found that CSF TMAO levels were notably higher in individuals with MCI and AD dementia compared with those without cognitive impairment [34]. In this scenario, TMAO has been identified as the most predictive biomarker for memory impairment and cognitive decline among 56 microbiota-derived metabolic markers [123].

### 4.2. Parkinson’s Disease

Emerging evidence suggests that the cellular effects exerted by TMAO, previously discussed in Section 2, may contribute to the pathophysiology of Parkinson’s disease (PD) [133]. Activation of the NLRP3 inflammasome increases pro-inflammatory cytokine levels (IL-1β, IL-6, and TNF-α), which are implicated in dopaminergic neurodegeneration [134]. Another key mechanism previously outlined is TMAO-induced mitochondrial dysfunction, primarily via SIRT3-SOD2 inhibition. This disruption leads to excessive ROS production, oxidative stress [39], and α-synuclein aggregation, a hallmark of PD progression [135]. Therefore, the imbalance in mitochondrial homeostasis may be directly associated with synaptic dysfunction, which clinically manifests as a gradual decline in motor function. The observed association between TMAO and endothelial dysfunction, together with platelet hyperreactivity, could further contribute to cerebrovascular alterations, possibly worsening BBB integrity and reducing cerebral perfusion, conditions that have been linked to cognitive decline and dementia in Parkinson’s patients [136].

Furthermore, TMAO-driven monocyte and macrophage activation, as discussed in Section 2.4, may contribute to chronic low-grade inflammation, affecting both the central nervous system and peripheral organs [137]. Interestingly, TMAO has been shown to stabilize α-synuclein under physiological conditions, whereas lower TMAO levels may promote the accumulation of misfolded α-synuclein, suggesting a hormetic effect [138].

In line with this evidence, recent studies have reported elevated plasma TMAO levels in PD patients, with a strong correlation to disease severity and the progression of motor symptoms [33]. This has been demonstrated by evidence that baseline plasma TMAO concentrations are linked to a more rapid increase in the need for dopaminergic treatment and a higher risk of PD-related dementia, highlighting its potential as a prognostic biomarker [139]. Nonetheless, studies analyzing bacterial metabolites, including TMAO, short-chain fatty acids, isovalerate, succinate, and lactate, in the plasma of PD patients (both treatment-naïve and treated) and control groups revealed an upregulation of the TMAO pathway in the PD patients, independent of medication, disease characteristics, or lifestyle factors. Additionally, the ratio of pro-inflammatory TMAO to the potentially anti-inflammatory butyric acid was significantly higher in the PD patients than in the controls, suggesting a shift toward a pro-inflammatory metabolic profile [140].

### 4.3. Amyotrophic Lateral Sclerosis

Amyotrophic lateral sclerosis (ALS), also known as Lou Gehrig’s disease, is a progressive neurodegenerative disorder that affects the nerve cells responsible for controlling voluntary muscles. The precise cause of ALS remains unclear, but it is believed to result from a combination of genetic and environmental factors. These may include intestinal dysbiosis, the overproduction of certain metabolites by specific intestinal strains such as TMA and TMAO, and elevated plasma formaldehyde levels [141].

**Figure 2:**
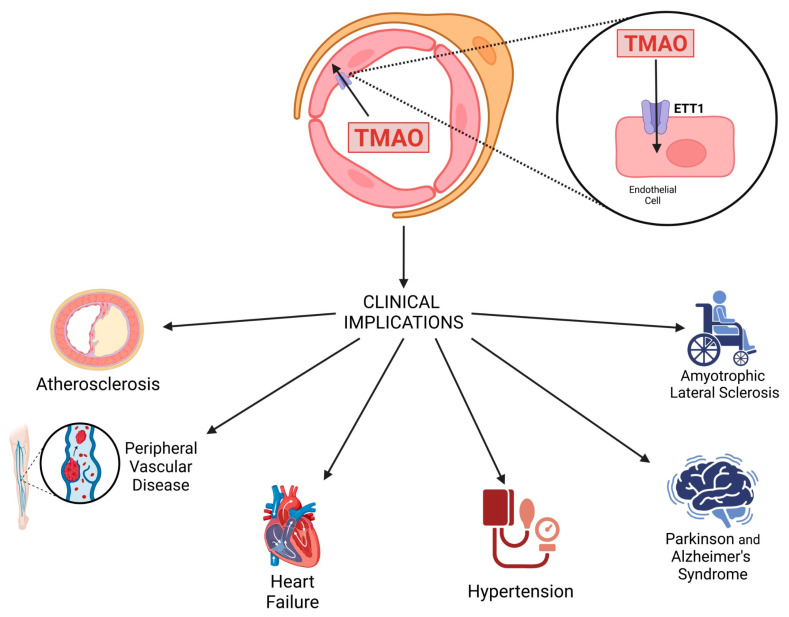
Schematic representation of TMAO transport into endothelial cells via the endothelial TMAO transporter (ETT) and its clinical implications.

The study conducted by Chen et al. examined the plasma concentrations of TMAO and its precursors in patients with ALS, comparing these concentrations with those in healthy controls and the spouses of the ALS patients. The results revealed that the ALS patients exhibit elevated levels of carnitine and betaine but reduced levels of choline, TMAO, and butyrobetaine compared with those of the healthy controls. Interestingly, the concentrations of TMAO and its precursors in the plasma of the ALS patients’ spouses were more similar to those in the ALS patients than to those in the healthy controls, suggesting disturbances in the gut microbe-mediated metabolism of choline and carnitine in the spouses, despite their healthy status [142]. These findings imply that alterations in the gut microbiota may precede the onset of ALS. A graphical representation of TMAO transport into endothelial cells and its clinical effects is provided in Figure 2.

## 5. Current Strategies to Manage TMAO Levels

Since TMAO is considered a potential high-risk factor for cardiovascular and neurological diseases, greater attention has to be paid to its prevention and treatment [143]. TMAO levels are directly related to the variety of consumed foods; therefore, diet plays a crucial role in reducing plasma TMAO levels. The basic principles include a reduction in the consumption of red meat; a change in diet to one rich in fruits, vegetables, whole grains, and legumes; and the use of cooking methods such as boiling or stewing instead of grilling, which can produce higher amounts of TMAO [144,145]. Notably, research indicates that vegetarians tend to have lower TMAO concentrations than omnivores [54]. Accordingly, a study comparing individuals on a standard diet with increased vegetable intake (VEG group) and those following a five-day low-calorie regimen (FMD group) found that intermittent fasting significantly decreased plasma TMAO [146]. Although the Mediterranean diet includes foods that naturally contain TMAO, studies suggest that these foods do not contribute to increased plasma TMAO levels in healthy individuals and may even reduce them by positively modifying gut microbiota [147]. However, it is important to note that dietary interventions alone may not be sufficient to reduce TMAO levels within physiological limits.

Probiotics have emerged as a potential alternative for modulating gut microbiota and mitigating atherosclerosis progression. Studies suggest that Lactobacillus plantarum ZDY04 could help reduce TMAO concentrations and prevent TMAO-induced atherosclerosis in animal models [148]. Other probiotic strains, including Bifidobacterium breve Bb4 and Bifidobacterium longum BL1 and BL7, have also demonstrated significant reductions in both plasma and cecal TMAO levels [149]. Additionally, Bifidobacterium animalis subsp. lactis F1-3-2 appears to decrease TMA levels in the cecum and improve lipid metabolism by regulating cholesterol 7-alpha hydroxylase and farnesoid X receptors [150]. Despite promising findings, probiotic interventions have yielded inconsistent results. For instance, a study testing the multi-strain probiotic VSL#3 found no significant impact on plasma TMAO levels or the concentrations of L-carnitine, betaine, or choline [151]. The varying capacity of different probiotics to metabolize choline may account for these conflicting outcomes.

Currently, no medications specifically designed to reduce blood TMAO levels exist, although ongoing research is being conducted to identify substances capable of bringing TMAO levels within physiological limits. Some medications have shown abilities to reduce TMAO levels as a side effect of their primary action, while others can increase TMAO levels as their main means of therapy. As an example, a review published in 2025 has highlighted the potential role of statins in lowering TMAO levels independently of their cholesterol-lowering effects [152]. Statins may exert this effect through multiple mechanisms, including alterations in bile acid metabolism, the suppression of TMA-producing bacteria, and the modulation of gut microbiota composition. Specifically, statins appear to downregulate the hepatic enzymes CYP7A1 and CYP27A1, which are involved in bile acid synthesis [153]. This reduction in bile acids indirectly influences gut microbiota, suppressing firmicutes and other TMA-producing bacteria, thereby reducing TMAO production [154]. Additionally, scientific evidence suggests that statins selectively inhibit the growth of pathogenic bacteria, such as Clostridium and Ruminococcus, while promoting beneficial species, such as Bifidobacterium and Lactobacillus [155]. In this scenario, clinical studies demonstrated that high-dose atorvastatin (80 mg) or the combination of atorvastatin/ezetimibe or rosuvastatin can significantly reduce TMAO levels, likely due to their direct action on the TMAO-producing gut flora [156,157]. However, chronic statin use, particularly at high doses, is associated with common side effects. Interestingly, the therapeutic efficacy of statins, measured by the HDL/LDL ratio, has been found to correlate directly with plasma TMAO levels. This relationship suggests that monitoring TMAO levels could help optimize therapy, enhance risk stratification, and lower the incidence of major cardiovascular events [157].

Loop diuretics, such as furosemide, are fundamental in the treatment of heart failure, although their prolonged use can worsen renal function and increase mortality [158]. Since they compete with the TMAO excretion mechanism, a direct relationship has been demonstrated between the use of loop diuretics, increased TMAO levels due to reduced excretion, and the onset of major cardiovascular events [111,159]. Analyzing the level of TMAO in this context could be of particular significance. The activation of the renin–angiotensin–aldosterone system (RAAS) is a crucial factor in the onset and progression of cardiovascular diseases, playing a protective role in maintaining renal function. ACE inhibitors help regulate cation and methylamine excretion by modulating organic cation transporters [160], which are particularly significant in TMAO excretion [161]. Consequently, these transporters may serve as potential biomarkers in hypertensive individuals with elevated TMAO levels [162].

Nonetheless, nutraceuticals have assumed a key complementary role in the management of TMAO levels [163]. In this context, resveratrol (3,5,4′ trihydroxystilbene, RSV) has been demonstrated to inhibit the pro-arteriosclerotic action induced by TMAO by reducing its levels through gut microbiota remodeling and increasing hepatic bile acid synthesis [164]. RSV is also a potent activator of sirtuins, capable of counteracting TMAO’s inhibitory action on mitochondrial sirtuins and reducing cellular inflammasome activation [165]. Nonetheless, quercetin, a flavonoid with excellent antioxidant properties, can reduce TMAO levels, preserving liver hepatocytes from its toxic action [166].

Based on this evidence, nutraceutical formulations containing the synergistic combination of bioactive molecules with TMAO-reducing potential could be particularly useful. In this regard, a grape pomace extract (Vitis vinifera L, cultivar Aglianico), microencapsulated with maltodextrins to increase its absorption, demonstrated a reduction in TMAO levels by around 60%, along with a similar reduction in LDL-OX, in both healthy individuals with a protein-rich diet and overweight individuals [167,168]. This formulation, known as Taurisolo^®^, contains high concentrations of polyphenols (e.g., resveratrol, catechins, quercetin, and quercetin-3-O-glucoside), which act synergistically to protect various target organs from elevated TMAO levels, including the endothelial cell wall of blood vessels involved in inflammatory processes [169]. As an example, a recent animal-based study demonstrated the protective effect of Taurisolo^®^ on brain microcirculation, particularly in the context of ischemia–reperfusion injury. The nutraceutical formulation, administered either intravenously or orally, significantly mitigated the microvascular damage associated with cerebral blood flow reduction and restoration, accompanied by increased eNOS expression and NO bioavailability [170]. Nonetheless, a more recent study showed the significant benefits of Taurisolo^®^ supplementation in diabetic patients with peripheral artery disease (PAD), a condition associated with high TMAO levels and endothelial dysfunction. After six months of treatment, their pain-free walking distances improved by 14.1%, while their serum TMAO levels dropped significantly from 3.97 to 0.87 µmol/L (*p* < 0.0001). Notably, these benefits persisted even after treatment discontinuation, indicating a continuing impact [114].

Altogether, these findings strongly support the therapeutic potential of nutraceutical formulations enriched with a variety of polyphenols in an appropriate balance, particularly under conditions where TMAO reduction and endothelial protection are essential for disease prevention and management.

## 6. Conclusions

The metabolite TMAO has emerged as a rising star in the field of metabolites, playing a pivotal role in the intricate interplay between diet, gut microbiota, and human health. Its increasing recognition as a biomarker for various diseases, including cardiovascular and neurodegenerative conditions, underscores its significance in clinical research and disease prevention. Research has shown that elevated TMAO levels contribute to endothelial dysfunction, inflammation, and atherosclerosis, highlighting the necessity of effective strategies aimed at regulating its levels. As a modifiable risk factor, TMAO levels can be influenced through dietary interventions, gut microbiota modulation, and targeted nutraceutical approaches. Future research should focus on elucidating the molecular pathways through which TMAO contributes to disease progression, with an emphasis on its interactions with host genetics, gut microbiota composition, and other metabolic pathways. Large-scale longitudinal studies and clinical trials are essential to establish causality between TMAO levels and disease outcomes, as well as to assess the efficacy of targeted interventions. Additionally, investigating novel therapeutic approaches, including probiotics, prebiotics, dietary modifications, and pharmacological agents, could provide promising strategies for mitigating TMAO-associated risks. From a clinical perspective, incorporating a TMAO assessment into routine risk stratification models may enhance the prediction and prevention of cardiovascular and metabolic disorders.

## Figures and Tables

**Table 1:** Effects of TMAO on various cell types and their molecular mechanisms.

Cell Type	TMAO Effect	Cellular Mechanism	Interaction Site	Reference
Monocytes	Increased pro-inflammatory monocyte levels (CD14++CD16+)	Not fully understood	-	[76]
Macrophages	Induction of endoplasmic reticulum (ER) stress	Activation of Toll-like receptor 4 (TLR4)	ER, TLR4	[77]
	M1 macrophage polarization	Activation of NLRP3 inflammasome	NLRP3	[84,85]
	Foam cell formation	Increased oxidized LDL cholesterol uptake and reduced cholesterol efflux	-	[50]
Platelets	Increased platelet aggregation	Altered Ca^2+^ signaling	-	[80]
Endothelial Progenitor Cells (EPCs)	Reduced EPC number and function	Inactivation of Akt/eNOS and MAPK/ERK signaling pathways	Akt/eNOS, MAPK/ERK	[71,72]
	Increased inflammation and oxidative stress	Increased microRNA-221 (miR-221) expression	miR-221	[72]
	Impaired neovascularization	-	-	[60,86]
Endothelial Cells	Endothelial dysfunction	Increased expression of adhesion molecules such as VCAM-1	VCAM-1	[87]
		Increased production of reactive oxygen species (ROS)	-	[41,87]
		Increased production of pro-inflammatory cytokines	NF-κB	[76,87]

## Data Availability

The data used to support the findings of this study are included within this article.
